# ^68^Ga-PSMA-11 PET/CT in restaging castration-resistant nonmetastatic prostate cancer: detection rate, impact on patients’ disease management and adequacy of impact

**DOI:** 10.1038/s41598-020-58975-8

**Published:** 2020-02-07

**Authors:** Aloÿse Fourquet, Cyrielle Aveline, Olivier Cussenot, Gilles Créhange, Françoise Montravers, Jean-Noël Talbot, Mathieu Gauthé

**Affiliations:** 1 Department of Nuclear Medicine, Hôpital Tenon-AP-HP, Sorbonne Université, Paris, France; 2 Department of Urology, Hôpital Tenon, AP-HP, Sorbonne Université, Paris, France; 3 Department of Radiation Therapy, CLCC Georges-François Leclerc, Dijon, France; 4 Department of Radiation Therapy, Institut Curie, Saint-Cloud, France; 5 AP-HP Health Economics Research Unit, INSERM-UMR1153, Paris, France

**Keywords:** Prostate cancer, Cancer imaging, Prostate

## Abstract

We aimed to evaluate the impact of prostate-specific membrane antigen ligand labelled with gallium-68 (PSMA-11) PET/CT in restaging patients with castration-resistant nonmetastatic prostate cancer (PCa). Thirty patients were included. At least one malignant focus was found in 27/30 patients (90%). The PSMA-11 PET/CT positivity rate in patients whose prostate-specific antigen serum level (PSA) was greater than 2 ng/ml was 100% (20/20), significantly superior to that of patients whose PSA was less than 2 ng/ml (7/10 = 70%). Six patients (20%) were categorized as oligometastatic (≤3 metastatic foci). Based on the 17 patients for whom a standard of truth was feasible, the overall sensitivity and specificity of PSMA-11 PET/CT in detecting residual disease in castration-resistant PCa patients were 87% and 100% respectively. PSMA-11 PET/CT impacted patients’ disease management in 70% of cases, 60% of case when PSA was less than 2 ng/ml. This management was considered as adequate in 91% of patients. PSMA-11 PET/CT appeared to be effective in restaging patients with castration-resistant nonmetastatic PCa. PSMA-11 PET/CT should be considered as a replacement for bone scans under these conditions.

## Introduction

Castration-resistant prostate cancer (CRPC) is defined by a castrate serum testosterone less than 50 ng/dl plus a biochemical progression (three consecutive rises in prostate-specific antigen (PSA) one week apart, resulting in two 50% increase over the nadir, and a PSA greater than 2 ng/ml) and/or a radiologic progression (appearance of new lesions according to the Response Evaluation in Solid Tumors criteria)^1^. Progression to castration-resistance is part of the natural evolution of prostate cancer (PCa) patients under androgen-deprivation therapy (ADT). The incidence rate of castration-resistance is estimated to be 8.3 cases per 100 people per year in castrated PCa patients^2^.

The use of systemic ADT to treat asymptomatic nonmetastatic recurrent PCa patients who are not eligible for curative treatment is controversial, as the benefit of this therapy for patients’ disease management remains unclear compared to its risk of side effects^3^.

The prostate-specific membrane antigen (PSMA) is a transmembrane protein that is over-expressed up to 1000 times by almost all PCa cells^4,5^. The recent introduction of positron emission tomography associated with computed tomography (PET/CT) using a radioligand of PSMA for PCa imaging has had an impact on the therapeutic management of PCa patient with biochemical recurrence (BCR)^6–8^, but its interest in restaging CRPC patients is unclear. PSMA over-expression was demonstrated to be higher in CRPC and as an effect of ADT in hormone naïve PCa^5,9,10^. Thus, this imaging modality may also influence the management of CRPC patients, in particular by upstaging them from nonmetastatic to metastatic and so triggering stereotactic radiation therapies or introduction of second-generation ADT^11^. Recently, the notion of oligometastatic PCa has been revisited^12^. Therefore, PET/CT was highlighted in oligometastatic PCa patients as it allows targeted therapies with curative intent of detected abnormal foci^13^.

In this work, we aimed to evaluate the usefulness of PET/CT using a ligand of PSMA radiolabeled with gallium-68 (^68^Ga) (PSMA HBED CC or PSMA-11) in restaging PCa patients upon the onset of resistance to castration, its impact on patients’ disease management and the adequacy of this impact.

## Results

### CRPC patients’ characteristics

Thirty nonmetastatic PCa patients treated by ADT and referred for PSMA-11 PET/CT between June 2016 and November 2018 because of a rise in PSA despite a suitable castrate serum level of testosterone were retrospectively included in this study (Table 1). None were symptomatic. The median time from PCa diagnosis to the onset of resistance to castration was 82 months [range: 10–258]. The median time under ADT before PSMA-11 PET/CT was 38 months [range: 14–108]. Among the 10 patients whose PSA was less than 2 ng/ml, seven had a PSA doubling time under 12 months. Fifteen of the 22 operated patients had a second-line radiation therapy for BCR.

**Table 1:** Patient characteristics.

Parameter	
n	30
**Median age in years [range]**	
At prostate cancer diagnosis	61 [50–81]
The day of PSMA-11 PET/CT	71 [58–86]
**Initial group according to d’Amico classification**	
Low risk	1 (3%)
Intermediate risk	9 (30%)
High risk	16 (53%)
Unknown	4 (14%)
**International Society of Urological Pathologists (ISUP) 2014 grade group**	
1	3 (10%)
2	9 (30%)
3	5 (17%)
4	7 (23%)
5	5 (17%)
Unknown	1 (3%)
**Initial treatment**	
Surgery (prostatectomy ± lymph node dissection)	22 (73%)
Definitive radiation therapy ± androgen deprivation therapy	7 (23%)
Brachytherapy	1 (4%)
**Type of ongoing androgen deprivation therapy**	
Anti-androgen	3 (10%)
LHRH agonist	14 (47%)
CYP17 inhibitor	1 (3%)
Anti-androgen + LHRH agonist	11 (37%)
Anti-androgen + LHRH antagonist	1 (3%)
**PSA parameters at PSMA-11 PET/CT**	
Median serum level [range]	3 ng/ml [0.3–90]
Less than 2 ng/ml	10 (33%)
Greater than 2 ng/ml	20 (67%)
Median doubling time [range]	5.8 months [−10.1–12.2]
Under 6 months	15 (50%)
Between 6 and 12 months	12 (40%)
Above 12 months	3 (10%)

Three patients were considered lost to follow-up (no visit to the referring clinician or no data available for evaluating the treatments’ efficacy after PSMA-11 PET/CT), but the impact of imaging was assessable for all of them because the multidisciplinary meeting report were available. The median duration of follow up after PSMA-11 PET/CT for the 27 assessable patients was 12 months (range 3–35). A SOT was feasible in 17/30 patients (57%) according to the criteria outlined above. Histological data were available for 5 patients.

### PSMA-11 PET/CT performances overall, per anatomical sites and according to PSA serum level

At least one malignant focus was found in 27/30 patients (90%). The overall and per anatomical sites positivity rates of PSMA-11 PET/CT, irrespective of PSA, for both routine unmasked and retrospective masked readings, as well as the median maximum standardized uptake value (SUVmax) of detected foci (measured during the retrospective masked reading) are presented in Table 2. The PSMA-11 PET/CT positivity rate in patients whose PSA was greater than 2 ng/ml was 20/20 = 100%, significantly superior to that of patients whose PSA was less than 2 ng/ml (7/10 = 70%; p = 0.01). PSMA-11 PET/CT was positive for 26/27 patients (96%) whose PSA doubling time was under 12 months versus for 1/3 (33%) patient whose PSA doubling time was above 12 months (p = 0.001).

**Table 2:** PSMA-11 PET/CT findings in nonmetastatic castration-resistant prostate cancer patients investigated because of a rise in prostate-specific antigen serum level value (irrespective of total prostate-specific antigen serum values).

n = 30	Malignant	Equivocal	Negative	SUVmax [range]	κ
**Overall**					
Routine unmasked	26	1	3		
Retrospective masked	27	0	3		0.52
**Prostate/prostatic lodge**					
Routine unmasked	10	1	19		
Retrospective masked	8	0	22	8.8 [3.7–124]	0.63
**Pelvic lymph nodes**					
Routine unmasked	16	0	14		
Retrospective masked	13	1	16	9.3 [1.3–25.7]	0.49
**Paraaortic lymph nodes**					
Routine unmasked	12	0	18		
Retrospective masked	12	0	18	8.5 [4.3–52.9]	1
**Lymph nodes above the diaphragm**					
Routine unmasked	9	1	20		
Retrospective masked	11	1	18	9.9 [2.7–54.2]	0.80
**Bone**					
Routine unmasked	8	1	21		
Retrospective masked	9	1	20	6 [2.3–48.4]	0.85
Viscera*					
Routine unmasked	4	0	26		
Retrospective masked	4	0	26	5.5 [4.9–11.4]	1

*two cases of lung metastases and 2 cases of peritoneal carcinomatosis.

The results of routine unmasked and retrospective masked readings, both by anatomical site and overall, are presented. Median maximum standard uptake values (SUVmax) per anatomical sites are presented with their range between brackets. Agreement evaluated with Cohen’s kappa coefficient κ.

Three patients presented both a PSA less than 2 ng/ml and a PSA doubling time above 12 months. PSMA-11 PET/CT imaging was positive for one of them, diagnosing a polymetastatic disease.

Based on the 17 patients for whom the SOT was feasible, the overall sensitivity and specificity of PSMA-11 PET/CT in detecting residual disease in CRPC patients were 87% and 100%, respectively, when considering the retrospective masked reading of the imaging.

According to PSMA-11 PET/CT results, 3 patients (10%) had no detectable disease (PSA of 0.3, 0.4 and 1.5 ng/ml), 2 patients (7%) presented an isolated focus in the prostate/prostatic lodge (PSA of 1.6 and 3 ng/ml), 6 patients (20%) were categorized as oligometastatic (range of PSA: 0.4–21 ng/ml) and 19 patients (63%) were polymetastatic (range of PSA: 0.9–90 ng/ml). In the 10 patients with a PSA less than 2 ng/ml, 3 had no detectable disease, one had an isolated malignant focus in the prostatic lodge, 3 were oligometastatic, and 3 were polymetastatic.

The dynamic images acquired on the pelvis immediately after PSMA-11 injection gave no additional diagnostic information, since all abnormal foci in the prostatic lodge were also detected on the vertex to mid-thigh acquisition performed 60 to 90 minutes after injection.

### Impact of PSMA-11 PET/CT and adequacy of CRPC patients’ disease management

Patients’ disease management changed in 26/30 (87%) patients; PSMA-11 PET/CT impacted this change in 21 cases (70%), among which 6 had a PSA less than 2 ng/ml (Table 3). The following changes occurred as a result of PSMA-11 PET/CT: 6 patients were given a stereotactic radiation therapy of the detected malignant foci (1 nonmetastatic, 4 oligometastatic and 1 polymetastatic), 14 patients (1 oligometastatic and 13 polymetastatic) underwent a second-generation ADT (abiraterone acetate or enzatulamide) and one polymetastatic patient was started on taxane chemotherapy.

**Table 3:** Impact on patient management: treatment scheduled before and in view of PSMA-11 PET/CT findings, overall and for PSA less than 2 ng/ml. Changes induced by PSMA-11 PET/CT results are highlighted in bold and underlined.

Management All PSA considered		Scheduled (n = 30)				
		No change in ADT n = 25	Change in ADT (excluding 2^nd^ generation ADT) n = 3	Introduction of 2^nd^ generation ADT n = 2	Chemotherapy n = 0	Stereotactic radiation therapy and no change in ADT n = 0
Indicated after PSMA-11 PET/CT	No change in ADT n = 4	3	1	0	0	0
	Change in ADT (excluding 2^nd^ generation ADT) n = 2	2	0	0	0	0
	Introduction of 2^nd^ generation ADT n = 17	**13** + 1	**1**	2	0	0
	Chemotherapy n = 1	**1**	0	0	0	0
	Stereotactic radiation therapy and no change in ADT n = 6	**5**	**1**	0	0	0
**Management** **PSA less than 2 ng/ml considered**		**Scheduled (n = 10)**				
		**No change in ADT** **n = 9**	**Change in ADT (excluding 2**^nd^ **generation ADT)** **n = 0**	**Introduction of 2**^**nd**^ **generation ADT** **n = 1**	**Chemotherapy** **n = 0**	**Stereotactic radiation therapy and no change in ADT** **n = 0**
Indicated after PSMA-11 PET/CT	No change in ADT n = 2	2	0	0	0	0
	Change in ADT (excluding 2^nd^ generation ADT) n = 1	1	0	0	0	0
	Introduction of 2^nd^ generation ADT n = 4	**3**	0	1	0	0
	Chemotherapy n = 0	0	0	0	0	0
	Stereotactic radiation therapy and no change in ADT n = 3	**3**	0	0	0	0

ADT: androgen-deprivation therapy; PSA: prostate-specific antigen.

In the 5 patients in whom the change in management was not motivated by PSMA-11 results, all were treated with a modification of ADT, including the introduction of a second-generation ADT for 3 of them. The 4 patients whose management was not changed kept their current ADT regimen.

Adequacy of management was assessed for 27 patients because 3 patients were lost to follow up. Patients’ disease management was considered adequate according to the defined criteria in 78% (21/27) of patients overall, 91% (19/21) when guided by PSMA-11 PET/CT versus 40% (2/5) when PSMA-11 PET/CT had no impact (p < 0.001).

### Agreement between routine unmasked and retrospective masked PSMA-11 PET/CT readings

The agreement between routine unmasked and retrospective masked readings, both overall and per anatomical site, is presented in Table 2. The overall agreement was moderate (k = 0.52). The agreement was moderate for pelvic lymph nodes (k = 0.49), strong for the prostate/prostatic lodge (k = 0.63) and lymph nodes above the diaphragm (k = 0.80), and very strong for bone (k = 0.85), paraaortic lymph nodes (k = 1) and viscera (k = 1).

## Discussion

**Figure 1:**
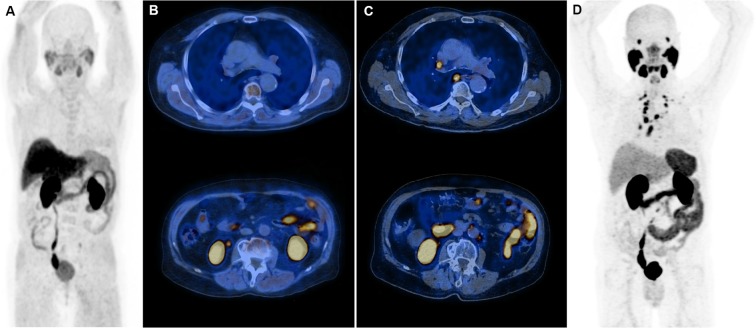
(**A**) 77-year-old man initially treated by prostatectomy 23 years before PSMA-11 PET/CT (ISUP 1, intermediate risk according to d’Amico classification). The first biochemical recurrence occurred 10 years after the surgery and was treated by radiation therapy of the prostatic lodge, which had no effect on PSA serum level, and androgen deprivation therapy (ADT) by LHRH agonist was initiated (PSA nadir undetectable). (**A**) rise in PSA appeared 1 year before PSMA-11 PET/CT. ^18^F-flurocholine PET/CT, which was performed 4 months before PSMA-11 PET/CT, was negative (PSA at 4.6 ng/ml) (**A**: maximum intensity projection view (MIP); (**B**): axial fusion slices centered on the thorax and the abdomen). PSMA-11 PET/CT (PSA at 5.5 ng/ml) demonstrated multiple malignant foci in the paraaortic region and in lymph nodes located above the diaphragm (**D**: MIP; **C**: axial fusion slices centered on the thorax and the abdomen), probably related to prostate cancer. A second generation ADT was started (PSA at 10 ng/ml) and was effective on PSA (at 4.8 ng/ml and stable 15 months after ADT modification).

PSMA is a known target for theranostics approach in PCa and various radiolabelled ligands of PSMA are available. PSMA-11 labelled with ^68^Ga is the most studied ligand for imaging PCa, especially PCa patients with BCR. Recent meta-analyses reported an overall positivity rate of 76% of ^68^Ga-PSMA PET/CT in PCa BCR^7,14^. This positivity rate varied from 45% for a PSA between 0.2–0.5 ng/ml to 95% for a PSA more than 2 ng/ml^7,14^. However, the usefulness of ^68^Ga-PSMA PET/CT in restaging CRPC and its impact on CRPC patients ‘management is unclear. In this study we aimed to fulfil this lack of data on a homogenous series of nonmetastatic PCa patients treated by ADT in whom a resistance to castration was developing, and whose recent ^18^F-fluorocholine PET/CT imaging was not able to detect residual metastatic PCa disease. We found an overall positivity rate of 90%, which was significantly superior to the 76% positivity rate that we previously reported in 33 PCa patients with a first BCR (p = 0.01, chi-squared test)^6^. This higher positivity rate is probably due to a higher expression of PSMA protein in CRPC tumour tissue as a result of ADT^5,10^. Consequently, a large majority of patients (25/30) were up-staged from nonmetastatic to metastatic CRPC according to PSMA-11 PET/CT results (Fig. 1).

**Figure 2:**
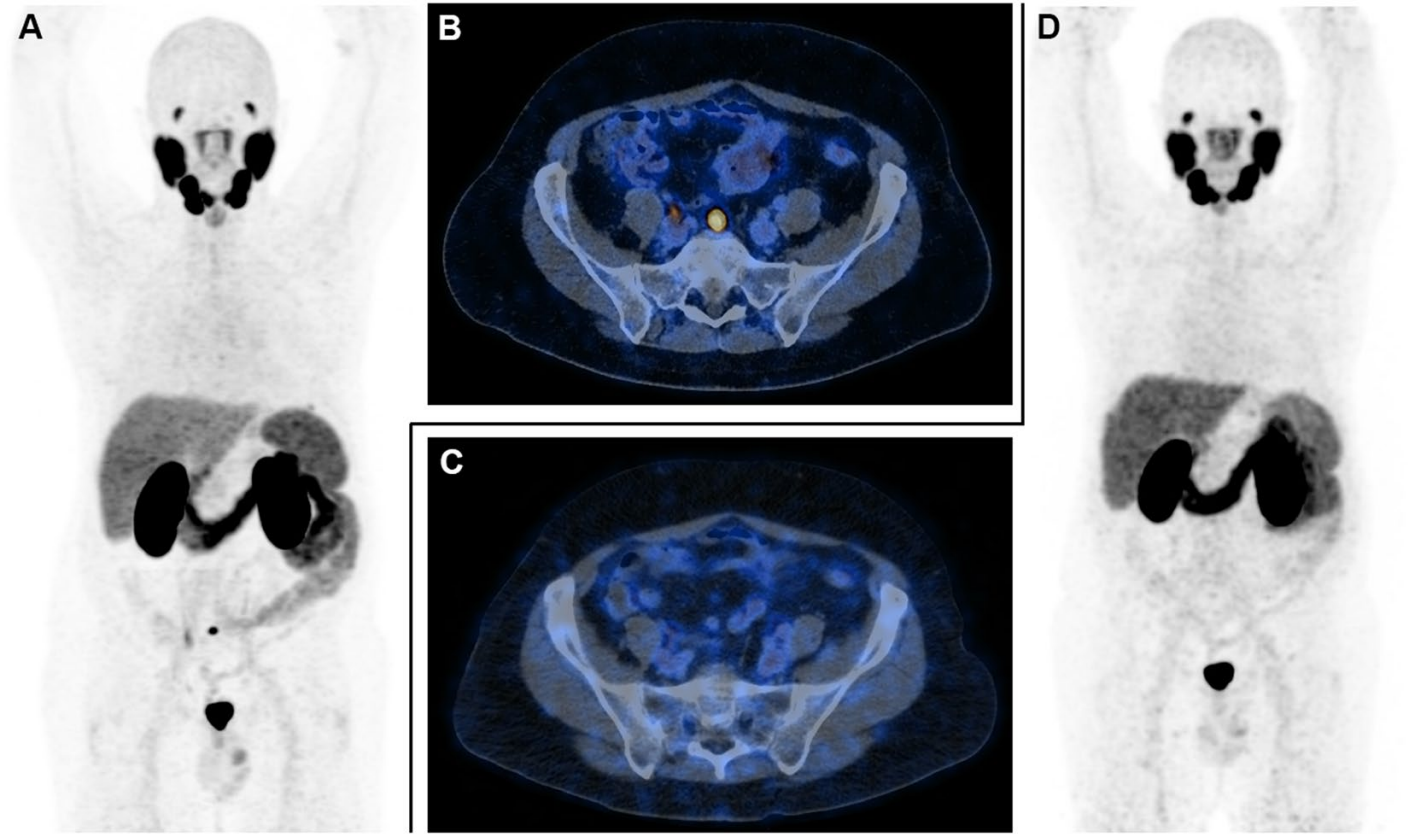
A 61-year-old man initially treated by prostatectomy 6 years before the PSMA-11 PET/CT (ISUP 4, high risk according to d’Amico classification). The first biochemical recurrence occurred 20 months after the surgery and was treated by radiation therapy of the prostatic lodge, which had no effect on the PSA serum level, and androgen deprivation therapy (ADT) by LHRH agonist was initiated (PSA nadir undetectable). (**A**) Rise in the PSA appeared 3 years after the beginning of ADT. ^18^F-flurocholine PET/CT, which was performed 3 months before PSMA-11 PET/CT, was negative (PSA at 1.3 ng/ml). PSMA-11 PET/CT (PSA at 1.5 ng/ml) demonstrated an isolated malignant focus in a presacral lymph node (**A**: maximum intensity projection view (MIP); (**B)**: axial fusion slices centered on the pelvis), probably related to prostate cancer. Stereotactic radiation therapy was performed on the presacral lymph node and was effective on PSA (at 0.28 ng/ml stable 12 months after PSMA-11 PET/CT). A second PSMA-11 PET/CT performed 10 months after the first one demonstrated the disappearance of the malignant locus from the presacral lymph node (**D**: MIP; **C**: axial fusion slices centered on the pelvis). The ADT regimen was not changed.

In our study, we deliberately did not take into account the complete definition of CRPC, which requires a PSA greater than 2 ng/ml in addition to a biochemical progression despite a suitable castrate serum testosterone^1^. We assumed that the occurrence of consecutive rises of PSA in PCa patients properly treated by ADT was enough to trigger an imaging workup, even if the PSA was less than 2 ng/ml. Our objective was to detect, thanks to PSMA-11 PET/CT, PCa residual disease early enough that there are only a small number of metastatic sites to propose a targeted treatment with curative intent to patients^15^. In our study, we found that the overall positivity rate of PSMA-11 PET/CT was rather important at 70% in patients presenting a PSA less than 2 ng/ml. This approach led to successful targeted treatment for 4/10 patients with a PSA less than 2 ng/ml (one patient with an isolated focus in the prostatic lodge and 3 oligometastatic patients) with stereotactic radiation therapy of the detected foci (Fig. 2). A fast PSA doubling time is associated with worse prognosis in nonmetastatic CRPC^1^. This parameter appeared relevant to predict PSMA-11 PET/CT positivity in CRPC patients, as at least one focus interpreted as malignant was found with PSMA-11 PET/CT in 96% of patients with a PSA doubling time under 12 months.

Current recommendations suggest that a bone scan should be performed in nonmetastatic CRPC patients when PSA reaches 2 ng/ml and should be repeated at higher PSA values if the first scan is negative^1,16^. The superiority of ^18^F-sodium fluoride PET/CT over bone scintigraphy for the diagnostic of PCa bone metastases was demonstrated more than 10 years ago^17^. More recently, a better specificity and an equal sensitivity of ^18^F-fluorocholine PET/CT compared to ^18^F-sodium fluoride PET/CT in the detection of PCa bone metastases were reported^18,19^. ^18^F-fluorocholine PET/CT has also been highlighted to be accurate in detecting PCa bone metastases, presenting a large concordance with bone scintigraphy or CT in CRPC patients^20^. Another study demonstrated that ^18^F-fluorocholine PET/CT detected more bone metastases than MRI of the spine in CRPC patients^21^. Therefore, we thought that a ^18^F-fluorocholine PET/CT negative for metastasis was sufficient to consider CRPC patients as nonmetastatic prior to PSMA-11 PET/CT restaging, without the need of another imaging modality.

As far as we found a 100% positivity rate when PSA was greater than 2 ng/ml, we propose that PSMA-11 PET/CT should replace bone scans for staging CRPC patients. Furthermore, PSMA-11 PET/CT should also be considered if the PSA is less than 2 ng/ml or if the PSA doubling time is under 12 months.

A recent meta-analysis reported a pooled proportion of patient management changes of 54% after ^68^Ga-PSMA PET/CT in 1163 PCa patients, none being precisely categorised as CRPC^8^. These changes resulted in an increased proportion of treatments with curative intent and a decreased proportion of ADT and surveillance^8^. In our study we found that PSMA-11 PET/CT impacted patients’ disease management changed in 70% of cases. We assume this higher impact of PSMA-11 PET/CT was related to the higher positivity rate of the imaging in CRPC patients. Moreover, PSMA-11 PET/CT results tended to trigger more treatments with curative intent in patients whose PSA was less than 2 ng/ml.

We determined the management adequacy based on the PSA serum evolution by taking into account the guidelines of the PSA working group^22^. Thus, a decrease in PSA of 50% from baseline was considered a PSA response if imaging, when available, showed no evidence of disease progression. Using these criteria, we found that patients’ disease management was statistically more effective when guided by PSMA-11 PET/CT (p < 0.001). To the best of our knowledge, such results have never been reported.

In this study, we found an overall moderate agreement between PSMA-11 PET/CT routine unmasked and retrospective masked readings (k = 0.52) by using a 3-point scale assessment. This agreement was 0.63 for the prostate/prostatic lodge, comparable to that was previously reported on a heterogeneous series of 50 patients at primary staging (n = 10), BCR (n = 25) and restaging for known metastatic disease (n = 10)^23^. Fendler *et al*. also reported a substantial to almost-perfect reproducibility for lymph nodes (k = 0.74, considering all lymph node areas)^23^, similar to the k = 0.71 of the present study. Furthermore, we chose to distinguish the invasion of the lymph nodes between the pelvic and paraaortic regions and above the diaphragm as we assumed that therapeutic management for involved lymph nodes differed between these areas. Interestingly, we found that reading agreement was strong and very strong for lymph nodes above the diaphragm (k = 0.80) and paraaortic lymph nodes (k = 1) respectively, but moderate for pelvic lymph nodes (k = 0.49). This result for pelvic lymph nodes might be explained by the risk of post inflammatory uptake in lymph nodes which was already reported^23^, as 73% (22/30) of the patients in our study had a history of radiation therapy of the pelvis for PCa. So, the experienced reader, even masked of clinical data, might have been more able to avoid this pitfall. Finally, we found a very strong agreement in bone staging (k = 0.85), which is comparable to that which was previously reported^23,24^.

Despite the disagreements in some of these results between readings for interpretation of PSMA-11 PET/CT, we simulated that the patients’ disease management could have differed only in 1/30 patient (3%) if the results of retrospective masked reading had been taken into account for decision making instead of the on-site unmasked reading: specifically, an isolated left supraclavicular lymph node was detected only upon retrospective reading, and therefore, stereotactic radiation therapy could have been discussed.

This study has several limitations. The main one, shared by most imaging studies addressing search for metastatic disease, was the lack of histological proof for most the suspected metastases, which were mainly characterized based on the follow-up data. We chose to base our SOT on the variation of PSA, excluding patients with a change in their ADT regimen after PSMA-11 PET/CT. In this work, a SOT was feasible for 57% of patients, with a 12-month median duration of follow up, which allowed us to calculate overall performances of PSMA-11 PET/CT. We decided to not calculate performances per anatomical region, as there was not enough abnormality per site to have relevant data. The second major limitation of this work was its retrospective design, the limited number of cases and the relative long inclusion period due to the rarity of PCa patients explored by PSMA-11 PET/CT in the setting of nonmetastatic PCa patients under ADT presenting a rise in PSA. However, our study was the largest homogenous study ever reported, and we found that restaging nonmetastatic CRPC patients by PSMA-11 PET/CT resulted in successful stereotactic radiation therapy of the detected lesions in 20% (6/30) of the patients, including 5 oligometastatic extensions.

The PSMA-11 PET/CT acquisition time varied in our study from 60 to 90 minutes after PSMA-11 injection, which may seem important as increased lesion detection was reported with delayed imaging times up to 4 hours^25^. However, acquisition times were within the acceptable range of 50 to 100 minutes which are recommended by the current guidelines for ^68^Ga-PSMA PET/CT^25^. Thus, we assume that the limited variation in acquisition times in our study did not affect the results significantly. Additionally, it is noteworthy that the dynamic acquisition performed over the pelvis immediately after PSMA-11 injection brought no additional information in this series and could be skipped in future.

Because of these limitations, the promising performances and impact rate on CRPC patients’ disease management of PSMA-11 PET/CT needs to be confirmed by a larger prospective study.

PSMA-11 PET/CT appeared to be effective in restaging nonmetastatic PCa patients treated by ADT and developing a resistance to castration, even when PSA was less than 2 ng/ml. in this context, PSMA-11 PET/CT motivated disease management changes in 70% of patients. Comparison between routine unmasked and retrospective masked readings showed it was highly reproducible, especially for detection of metastases in the paraaortic lymph nodes, lymph above the diaphragm and bone. As it also allows the detection of metastases in the soft tissues, PSMA-11 PET/CT should be considered as a replacement for bone scans in this setting.

## Methods

### Population

This retrospective study was composed of patients with PCa currently undergoing ADT who were referred in our department of nuclear medicine for a PSMA-11 PET/CT because of an increase in PSA despite a suitable castrate serum testosterone. These patients have also previously been diagnosed negative for metastasis based on routine imaging.

Inclusion criteria for patients were as follows: 1- histologically confirmed PCa initially treated with curative intent (radical prostatectomy, definitive radiation therapy, brachytherapy); 2- ADT was secondarily introduced because of a BCR; 3- no known history of PCa distant metastases (invaded locoregional pelvic lymph node at diagnosis was not considered as a metastatic status according to 2009 TNM classification for staging PCa^26^), the nonmetastatic status being confirmed by a ^18^F-fluorocholine PET/CT performed less than 3 months before PSMA-11 PET/CT; 4- currently presenting a biochemical progression defined as three consecutive rises in PSA, one week apart, resulting in two 50% increases over the nadir level, despite a castrate serum testosterone <50 ng/dl.

Exclusion criteria were as follows: 1- PCa with known distant metastases; 2- patients who were never treated with curative intent for PCa; 3- patients presenting a castrate serum testosterone ≥50 ng/dl; 4- the presence of another active neoplasm other than PCa.

The type of ongoing ADT (anti-androgen, CYP17 inhibitor, LHRH agonist or LHRH antagonist), the PSA and the PSA doubling time before the PSMA-11 PET/CT were noted.

According to French regulation, the approval of an institutional review board was not necessary for performing this retrospective analysis of already available data. However, the patients gave their written consent for the subsequent use of their PET/CT images for research purposes. PSMA-11 PET/CTs were performed as a compassionate use authorized on an individual basis by the National Medicine Agency.

### ^68^Ga-PSMA-11 PET/CT imaging procedure

^68^Ga was obtained from a ^68^Ge/^68^Ga radionuclide generator (GalliaPharm Eckert & Ziegler Radiopharma GmBH) and used for radiolabeling of the PSMA-11 according to the manufacturer’s instructions (IASON GmbH). The patients did not require specific preparation before the injection. Patients received 1–2 MBq/kg of the radiotracer, injected in saline via infusion line.

Images were acquired using a Gemini TF16 (Philips Healthcare) or a Biograph CTflow (Siemens Healthcare) PET/CTs. Dynamic images were acquired on the pelvis immediately after PSMA-11 injection (10 images of one-minute duration each) and from vertex to mid-thigh 60 to 90 minutes after injection. On the Gemini TF16 PET/CT, the pelvis was imaged for 3 minutes, and each other bed position was imaged for 2 minutes in 3D mode with a 576 mm FOV and a 144 × 144 matrix. Images were reconstructed from 3 iterations and 33 subsets using the OSEM weighted method. Low-dose CT without contrast-enhancement was performed prior to PET acquisition (120kVp, 80 mA.s, slice thickness 2.5 mm, pitch 0.813, rotation time 0.5 s, FOV 600 mm). On the Biograph CTflow PET/CT, the scanning speed was set to 0.7 cm/min over the pelvis and 0.9 cm/min for the rest of the acquisition field. Images were taken in 3D mode with a 780 mm FOV and a 200 × 200 matrix. Images were reconstructed from 2 iterations and 21 subsets using the OSEM weighted method. Low-dose CT without contrast-enhancement was performed prior to PET acquisition (CareDose® automatic modulation for keV and mA.s, slice thickness 2 mm, pitch 0.813, rotation time 0.5 s, FOV 500 mm).

### ^68^Ga-PSMA-11 PET/CT image analysis

The PSMA-11 PET/CTs were read on-site the day of the image acquisition (routine unmasked reading). A masked retrospective reading of the PSMA-11 PET/CTs of the patients matching the inclusion criteria was performed by an expert nuclear medicine physician who was masked to all clinical and biological data. Anonymized images presented in a random order were independently reviewed on a dedicated workstation (Syngo.via, Siemens Healthcare).

The masked reader evaluated foci across several anatomical sites and assigned them a value on a 3-point scale according to the uptake intensity: 0- no suspicious uptake (PSMA-11 uptake at best equal to muscles background); 1- equivocal uptake (PSMA-11 uptake between background in muscles and vessels); 3- malignant uptake (PSMA-11 uptake higher than background in vessels)^27^. CT images were used only for the anatomic allocation of a suspicious focus. Six anatomical sites were considered: prostate/prostatic lodge, pelvic lymph nodes (up to the common iliac lymph nodes), paraaortic lymph nodes, lymph nodes above the diaphragm, bone and viscera. An anatomical site was quoted as equivocal or malignant if at least one suspicious focus (equivocal or malignant) was detected in it. The SUVmax of the most intense abnormal focus was determined for each anatomical site. Patients were categorized as oligometastatic if between 1 and 3 distant malignant foci (excluding the prostate/prostatic lodge) were detected on PSMA-11 PET/CT; patients were categorized as polymetastatic if more than 3 distant malignant foci were detected^12^.

### Follow-up, standard of truth and impact of PSMA-11 PET/CT on patients’ disease management

Each patient was followed up by his referring physician after the imaging. The management plan was decided for each patient by clinicians during multidisciplinary meetings; the meetings before and after PSMA-11 PET/CT were considered for the purpose of this study. The multidisciplinary meeting panels were constituted by a urologist, a radiation oncologist, a pathologist and a PCa imaging specialist, who decided the management of the patients both pre and post PSMA-11 PET/CT. The impact of PSMA-11 PET/CT was defined as any change in management, during the multidisciplinary meeting, triggered by PSMA-11 PET/CT.

The existence of a PCa lesion was established for each patient according to a composite standard of truth (SOT) based on the PSA response (more than 50% compare to baseline value) to targeted PCa therapy (excluding change in ADT) and histological findings, if available. The management was considered to be adequate if the PSA declined by more than 50% (compared to the baseline value) following treatment modification or if the PSA remained stable (maximum variation of 10% compared to baseline) on at least 2 assays performed at least 3 weeks apart, when surveillance was decided^22^.

### Statistical analysis

IBM SPSS software was used for statistical calculations. Comparisons of PSMA-11 PET/CT detection rates to the PSA the day of the scan (less versus greater than 2 ng/ml, which is the threshold for the complete definition of CRPC^1^) and the PSA doubling time (under versus above 12 months, as this threshold is suggested for starting ADT in nonmetastatic CRPC^3^) were performed by a chi-squared test or a Fisher’s exact test according to the number of cases in each group. Adequacies of patients’ disease management when guided or not by PSMA-11 PET/CT imaging were assessed via Fisher’s exact test. A p value less than 0.05 was considered to be statistically significant. The agreement between retrospective masked and routine unmasked PSMA-11 PET/CT readings, overall and per anatomical site, were assessed using Cohen’s kappa coefficient (0–0.20: very weak; 0.21–0.40: weak; 0.41–0.60: moderate; 0.61–0.80: strong; 0.81–1.0: very strong). (Supplementary file 1).

## Supplementary information

STARD.

## Data Availability

The datasets generated during and/or analysed during the current study are available from the corresponding author on reasonable request.
